# Cardiorespiratory Phase-Coupling Is Reduced in Patients with Obstructive Sleep Apnea

**DOI:** 10.1371/journal.pone.0010602

**Published:** 2010-05-13

**Authors:** Muammar M. Kabir, Hany Dimitri, Prashanthan Sanders, Ral Antic, Eugene Nalivaiko, Derek Abbott, Mathias Baumert

**Affiliations:** 1 Centre for Biomedical Engineering, School of Electrical and Electronic Engineering, University of Adelaide, Adelaide, South Australia, Australia; 2 Cardiovascular Research Centre, School of Medicine, University of Adelaide, Adelaide, South Australia, Australia; 3 Thoracic Medicine Department, Royal Adelaide Hospital, Adelaide, South Australia, Australia; 4 School of Biomedical Sciences, University of Newcastle, Callaghan, New South Wales, Australia; 5 School of Medicine, School of Paediatrics and Reproductive Health, University of Adelaide, Adelaide, South Australia, Australia; University of Cape Town, South Africa

## Abstract

Cardiac and respiratory rhythms reveal transient phases of phase-locking which were proposed to be an important aspect of cardiorespiratory interaction. The aim of this study was to quantify cardio-respiratory phase-locking in obstructive sleep apnea (OSA). We investigated overnight polysomnography data of 248 subjects with suspected OSA. Cardiorespiratory phase-coupling was computed from the R-R intervals of body surface ECG and respiratory rate, calculated from abdominal and thoracic sensors, using Hilbert transform. A significant reduction in phase-coupling was observed in patients with severe OSA compared to patients with no or mild OSA. Cardiorespiratory phase-coupling was also associated with sleep stages and was significantly reduced during rapid-eye-movement (REM) sleep compared to slow-wave (SW) sleep. There was, however, no effect of age and BMI on phase coupling. Our study suggests that the assessment of cardiorespiratory phase coupling may be used as an ECG based screening tool for determining the severity of OSA.

## Introduction

Obstructive sleep apnea (OSA) is a disorder of breathing during sleep that affects over 4% of men and 2% of women [1]. Obstructive sleep apnea (OSA) is characterized by repetitive partial or complete closure of the upper airways that causes alterations in the functioning of cardiovascular and respiratory systems.

Cardiac autonomic nerve activity in OSA patients has been mainly studied using heart rate variability (HRV) methodology that is based on the assessment of ECG RR-interval changes [2]. Some indices of HRV were shown to be an independent predictor of cardiac mortality in different patient populations, including myocardial infarction [3], dilated cardiomyopathy [4], and congestive heart failure [5]. Autonomic modulation of the heart rate is altered during sleep in OSAS patients [6], [7], [8] and it has been proposed to use HRV as a screening tool [2], [9].

OSA patients have elevated sympathetic nerve activity [10], [11] and their HRV is altered [10], primarily by a great increase in very low frequency oscillations (VLF) that are caused by the heart rate bouts associated with repeated arousals [12]. The physiological mechanisms underlying HRV are not completely understood. Respiratory sinus arrhythmia is one of the main contributors to HRV. Spectral analysis of HRV typically reveals a high frequency (HF) component, which closely follows the respiratory frequency [13], [14] and is regarded as the most distinct feature of HRV [15].

Cardiorespiratory coordination is a concept based on physics that aims to quantify the interaction [16] between respiratory and heart rhythm, assuming they are generated by two independent systems. It was initially described as short intermittent periods [17], [18], [19] during which the phases of heart rate and respiratory rate coincide with different integer ratios known as phase locking ratios [20], [21], [22]. Cardiorespiratory coordination has been reported in healthy adults [22], [23], athletes [17], [20], sleeping humans [24], [25], infants [26] and anesthetized rats [27]. Although the mechanisms and physiological significance underlying coordination between respiration and heart rate are not understood, its quantification might have clinical merit e.g. estimating the prognosis of cardiac diseases after myocardial infarction in patients [28], [29].

This is the first study to explore cardiorespiratory coordination during sleep in a large cohort of patients with OSA. We hypothesize that cardiorespiratory coordination is reduced due to sleep apnea and its assessment provides markers of cardiorespiratory system disturbances.

## Methods

### 1. Ethics Statement

The study conformed to principles outlined in the Declaration of Helsinki and was approved by the local ethics committee, “Human Ethics Committee, Royal Adelaide Hospital”. Since de-identified data were collected from participants for this study, the ethics committee waived the need for written informed consents.

### 2. Subjects

Overnight sleep studies were performed in 248 patients (157 males/91 females) with suspected OSA. We excluded 35 patients with diabetes mellitus from this study due to suspected diabetic autonomic neuropathy that might potentially confound our results [30]. The age and BMI of the patients ranged 20–77 years (mean ± SD: 49.4±12.3 yrs) and 20.1–73.3 kg/m^2^ (mean ± SD: 34.1±8.14 kg/m^2^) respectively. In this cohort, 69 patients were reported to have cardiovascular disease. Initially, we separately analysed 144 patients without heart diseases and 69 patients with heart diseases and found no significant differences in the results between the two groups (see Table 1). Accordingly, the subsequent results were reported taking 213 subjects into account. However, the effect of age, gender and BMI were studied using linear regression model on the 144 patients without heart diseases.

**Table 1:** Percentage of coordination (%cordn) and duration of coordinated epochs (AvDurCordn) for different AHI groups and sleep stages using abdominal signal (ABDO) for patients with and without heart diseases.

			*AHI≤15*	*15<AHI<30*	*AHI≥30*
**Patients without heart disease**		Number	88	25	31
		Age (yrs)	43.1±10.6	51.7±12.4	43.0±8.9
		Male (%)	60.2	60	80.7
		BMI (kg/m^2^)	31.3±7.1	32.0±5.9	36.5±8.3
	**%cordn (%)**	SS1	12.1±6.1	10.1±5.3	6.7±4.4
		SS2	14.7±6.5	11.3±4.1	7.8±3.6
		SW	18.6±7.6	16.4±6.8	14.1±5.8
		REM	11.3±4.5	8.9±3.2	7.2±2.7
	**AvDurCordn (s)**	SS1	7.8±1.9	7.7±1.8	5.9±2.4
		SS2	8.6±1.6	8.6±2.1	6.3±2.1
		SW	9.1±1.6	9.4±1.8	7.9±2.2
		REM	7.7±1.8	7.4±1.9	6.1±1.7
**Patients with heart disease**		Number	35	15	19
		Age (yrs)	54.1±9.7	61.9±8.0	56.1±12.5
		Male (%)	48.6	73.3	68.4
		BMI (kg/m^2^)	34.6±10.2	32.4±2.4	41.8±8.3
	**%cordn (%)**	SS1	11.7±6.6	16.3±8.7	8.3±3.4
		SS2	15.0±6.2	12.3±5.4	9.0±4.6
		SW	18.9±9.3	16.1±9.0	15.1±6.3
		REM	11.0±4.0	10.4±4.7	6.8±4.1
	**AvDurCordn (s)**	SS1	7.8±2.3	7.6±2.1	6.3±2.2
		SS2	8.5±2.2	7.9±2.3	6.8±2.1
		SW	9.6±2.1	8.4±2.2	7.5±1.7
		REM	7.2±1.7	6.9±1.9	6.2±1.6

### 3. Data recordings and analysis

#### 3.1 Overnight polysomnography

Overnight polysomnography was performed using a E series® system (Compumedics, Australia). For sleep staging and arousal scoring standard surface electrodes were applied to the face and scalp, including two-channel electroencephalograms (EEG, C3-A2 and C4-A1), left and right electrooculograms (EOG) and a submental electromyogram. Leg movements were recorded from surface electrodes to tibialis anterior muscle of both legs. Respiratory depth and frequency was monitored using chest and abdominal respiratory inductance plethysmography bands. Sleep stages were assigned to consecutive 30 s epochs. Sleep scoring was carried out according to standard rules [31].

#### 3.2 ECG

The ECG signal (lead II) was digitized at 128 Hz and saved for off-line analysis. ECG R-wave peaks were detected using the programming library libRASCH (www.librasch.org). The RR intervals time series were visually scanned for artifacts and, if necessary, manually edited.

#### 3.3 Respiration

Abdominal and thoracic respiratory signals, digitized at 32 Hz, were used for the analysis of cardiorespiratory coordination. To remove noise, the signals were low-pass filtered at 0.5 Hz using a Zero-phase forward and reverse digital filter, which first filtered the raw signal in the forward direction using Butterworth filter, then reversed the filtered signal and subsequently filtered the reversed sequence. The resultant signal had precisely zero phase distortion which was examined by superimposing the filtered signal on the raw signal. Custom written computer software developed under MATLAB® was used to detect inspiratory onsets for each respiratory cycle. First, the offset of the signal was removed by subtracting its mean value. Subsequently, the inspiratory and expiratory onsets were determined as the zero-crossings of the first derivative of the respiratory signal. All zero-crossings less than 1.0 second apart were considered as artifact and hence discarded. The inspiratory onsets of respiration were later used to calculate the average respiratory time period.

#### 3.4 Cardiorespiratory coordination analysis

We used Hilbert transform to calculate the phases of the respiratory signal, and determined relationship between the respiratory phases at different R-peak instants. If we denote the phase of heartbeat as Φ_c_ and of respiratory signal as Φ_r_ and considering that the heart makes *m* heartbeats in *n* respiratory cycles, then phase coordination is defined as the locking of the corresponding phases given by
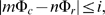
where *i* is a constant.

**Figure 1:**
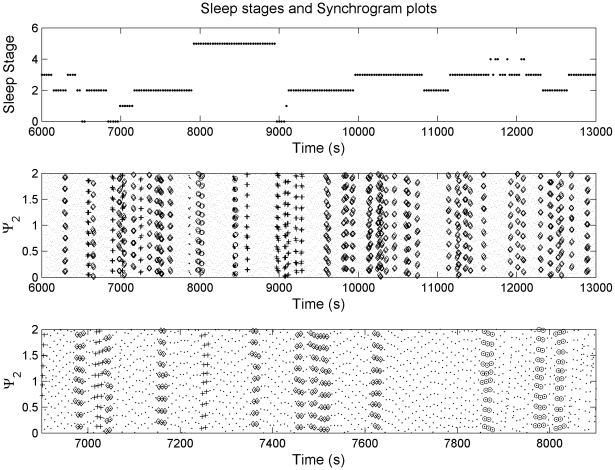
Synchrogram plot (second panel) revealing cardiorespiratory locking at different frequency ratios and sleep stages (rated at the top panel). Small dots indicate the normalised phases. Diamond, plus and circle indicates 9∶2, 10∶2 and 11∶2 coordination respectively. The numbers 0–5 for sleep stage represents awake, stage 1, stage 2, stage 3, stage 4 and REM sleep respectively. The recorded data segment for this figure was selected such that all the sleep stages are available in order to compare the differences. For better visualisation, an expanded time scale version of the synchrogram plot (second panel) has been shown in the third panel.

In other words, if the phase difference between the two oscillators was within a certain threshold value, *i* and remained stable for *n* respiratory cycles, the oscillators were considered coordinated. If *t_k_* is the time of the appearance of a *k* ^th^ R-peak, then by observing the phase of the respiration at the instants *t_k_*, denoted by Φ_r_(*t_k_*) and wrapping the respiratory phase into a [0, 2π*m*] interval, we can generate cardiorespiratory synchrogram. This provides a visual tool to detect cardiorespiratory coordination (Figure 1, second panel), by plotting Ψ*_n_* against *t_k_* which, in case of *m*∶*n* coordination, results in *m* horizontal lines. Here Ψ*_n_* is given by the equation
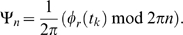



In order to determine the values of *m* and *n*, we selected one value of *n* at a time and looked for coordination at different values of *m*. The study was carried for the following *m*∶*n* coordination: *n* = 1: *m* = 2,…,8; *n* = 2: *m* = 5,7,9,11,13 and *n* = 3: *m* = 7,8,10,11,13,14,16,17,19,20. We used a threshold value of *i* = 0.025 for the phase difference as suggested by Cysarz *et al.* [24].

**Figure 2:**
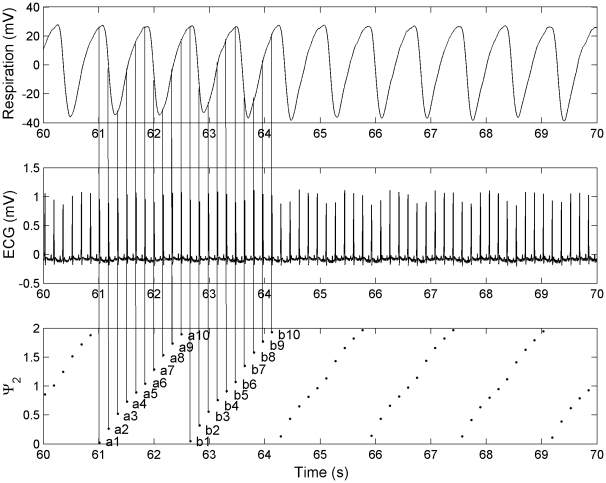
Illustration of the generation of synchrogram from the respiratory and ECG signals. a1, a2,…,a10 and b1, b2,….,b10 in the synchrogram plot represent the respiratory phases, based on the time points of R-peaks, for the first two and the following two respiratory cycles.

We presented an illustration (Figure 2) of how the synchrogram was generated from the respiratory and ECG signals. The phases of the respiratory signal, corresponding to the time points of the R-peaks, were plotted as normalized phases between 0 and 2. Subsequently, the phases for every two respiratory cycles formed a relatively vertical line: a1,a2,…,a10; b1,b2,…,b10; and so on. We then determined the differences between each point of one line to each corresponding point of the next line: a1-b1, a2-b2,…,a10-b10. If the differences between all the corresponding points were below the threshold value of 0.025, the respective R-peaks were considered as coordinated. In Figure 2 (third panel), if lines were drawn between the points a1∶b1, a2∶b2,…,a10∶b10, a structure of parallel horizontal lines, as termed by Cysarz *et al.* [24], would be observed. Similarly, from figure 2 (first panel), it could be observed that every two respiratory cycles consisted of ten equidistant R-peaks, indicating a phase locking ratio of 10∶2 (or 5∶1).

We employed two parameters for characterising cardiorespiratory coordination: Firstly, we calculated the percentage of coordination, %cordn, by adding up the time of all coordinated epochs observed in a particular segment or sleep stage and then dividing it by the total duration of the segments or sleep stage. Consequently, we computed %cordn_Total_ and %cordn_SleepStage_. Secondly, we measured the average duration of all coordinated epochs, AvDurCordn, for every sleep stage by calculating the arithmetic mean of the durations of coordinated epochs. Further, the phase locking ratio for each coordinated epoch was recorded and the total number of a particular locking ratio for every sleep stage was calculated (see Table 2 and 3) to obtain a histogram of different locking ratios.

**Table 2:** Number of *m*∶*n* coordinated epochs observed during awake and stage 2 (SS2) sleep.

		Awake			SS2		
	Respiratory cycle (*n*)	1	2	3	1	2	3
**RR cycle (** ***m*** **)**							
2		30			6		
3		27			29		
4		50			63		
5		26	5		46	4	
6		11			28		
7		3	23	0	3	28	0
8		1		2	0		6
9			14			35	
10				3			8
11			11	10		12	17
12							
13			2	12		2	18
14				3			4
15							
16				0			2
17				1			0

Each number represents the mean of the results obtained from 213 patients for the specific *m*∶*n* coordination as observed during a particular sleep stage.

**Table 3:** Number of *m*∶*n* coordinated epochs observed during slow-wave (SW) and rapid-eye-movement (REM) sleep.

		SW			REM		
	Respiratory cycle (*n*)	1	2	3	1	2	3
**RR cycle (** ***m*** **)**							
2		26			19		
3		31			21		
4		81			47		
5		47	7		35	3	
6		27			10		
7		12	24	0	2	6	0
8		0		3	0		5
9			22			5	
10				5			3
11			11	14		4	7
12							
13			4	9		2	6
14				4			2
15							
16				2			3
17				1			4

Each number represents the mean of the results obtained from 213 patients for the specific *m*∶*n* coordination as observed during a particular sleep stage.

#### 3.5 Surrogate data analysis

In order to determine whether randomness of heart rate variations play any role with respect to cardiorespiratory coordination in OSA patients, we used surrogate data for our analysis. The surrogate data were obtained by randomizing the order of RR intervals separately for every sleep stage and constructing the new R time series by starting with the first original R time and cumulating the randomized RR intervals. Accordingly, the phases of the respiratory signal corresponding to the new time points of the R-peaks were calculated and analysed for cardiorespiratory coordination.

#### 3.6 Statistical analysis

Statistics computer software SPSS, Inc. version 15.0 and GraphPad Prism, Inc. version 5.0 were used to analyse the data. To determine the effects of age, gender, and BMI on cardiorespiratory coordination, linear regression models were developed for the 144 patients without heart disease. To determine the effect of OSA severity on cardiorespiratory coordination, the cohort was trichotomized based on the apnea-hypopnea index (AHI): AHI≤15, 15<AHI<30, and AHI≥30. Differences in cardiorespiratory coordination between the three OSA groups as well as between different sleep stages were assessed with 2-way ANOVA for repeated measurements. For post-hoc analysis, Tukey's multiple comparison test was used. Values *p*<0.05 were considered statistically significant.

## Results

### 1. Polysomnographic findings

Demographic and polysomnographic data are presented in Table 4. Of the 213 patients, 133 (70 m) presented with no or mild OSA (AHI≤15), 40 (26 m) patients with mild to moderate OSA (15<AHI<30) and 50 patients (38 m) with severe OSA (AHI≥30). Neither age nor BMI were significantly different between the three subgroups.

**Table 4:** Demographic data and overnight polysomnography findings in different groups of OSA patients.

	*AHI≤15*	*15<AHI<30*	*AHI≥30*
Total number of patients, N_Total_	133	51	64
Patients with diabetes mellitus, N_DM_	10	11	14
Patients analysed, *N* (N_Total_−N_DM_)	123	40	50
Male	70	26	38
Age (yrs)	46±11	56±12	48±12
BMI (kg/m^2^)	32±8	32±5	39±9
Weight (kg)	91.2±21.4	93.4±19.6	111.2±23.6
Height (cm)	168.6±9.2	170.2±10.2	170.6±8.3
RR interval (ms)	911±120	956±131	845±123
Respiratory interval (ms)	3797.5±335.5	3947.4±315.8	3703.7±434.2
Total length of recording (min)	428.8±53.4	431.1±64.6	438.2±27.1
Sleep time (min)	346.0±55.4	346.9±60.6	352.8±64.8
Wake time (min)	61.1±42.4	64.7±42.3	74.1±56.8
REM sleep (%)	14.7±4.1	14.6±3.8	12.3±4.5
Arousals (#/h)	16.4±8.4*	25.3±8.6*	50.7±19.6*
Total percentage of coordination (%)	13.5±6.2*	11.6±5.1*	8.8±4.5*
Mean duration of coordinated epochs (s)	8.3±1.8*	8.1±2.0*	6.5±2.1*
Hypertension	26	14	18
Structural heart disease	18	8	6
Smoker	14	2	8

Data are presented as mean ± standard deviation. Astericks indicate statistically significant changes (*p*<0.001).

Sleep time, wake time and percentage of REM sleep were not significant between the three OSA subgroups (*p*>0.5). The number of arousals per hour was significantly higher in patients with severe OSA (50.7±19.6 #/h) compared to patients with moderate (25.3±8.6 #/h) or no/mild (16.4±8.4 #/h, *p*<0.0001) OSA,

Heart rate and respiratory rate were significantly elevated in patients with severe OSA compared to patients with no/mild OSA (Table 4).

**Figure 3:**
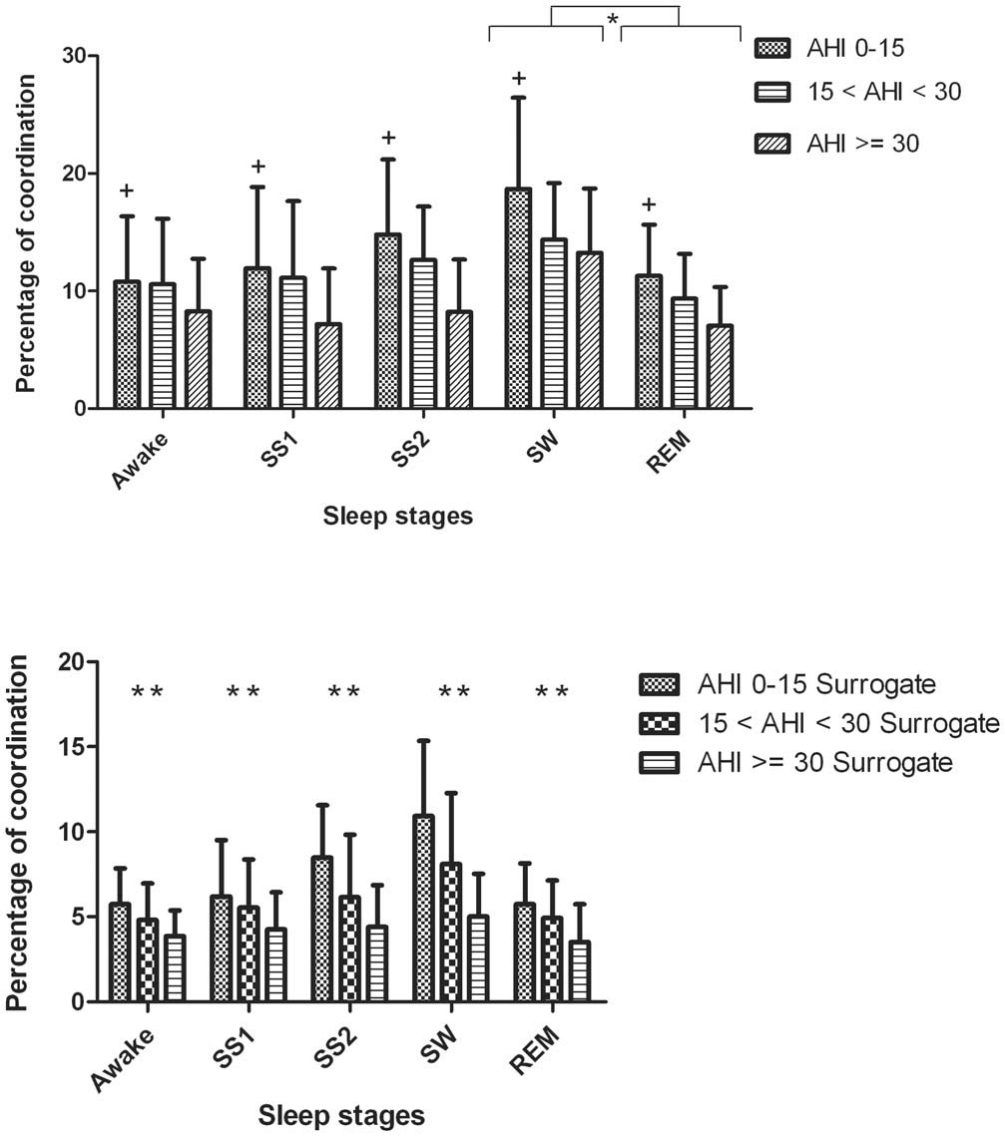
Group and sleep related comparison of percentage of coordination (%cordn, mean ± SD) using original (upper panel) and surrogate data (lower panel) from abdominal signal. The plus indicates that the differences in %cordn between the no/mild and severe OSA groups are significant (*p*<0.05). * (*p*<0.01) and ** (*p*<0.001) indicates the significant differences in %cordn between the SW and REM sleep stages and between the original and surrogate data respectively.

**Figure 4:**
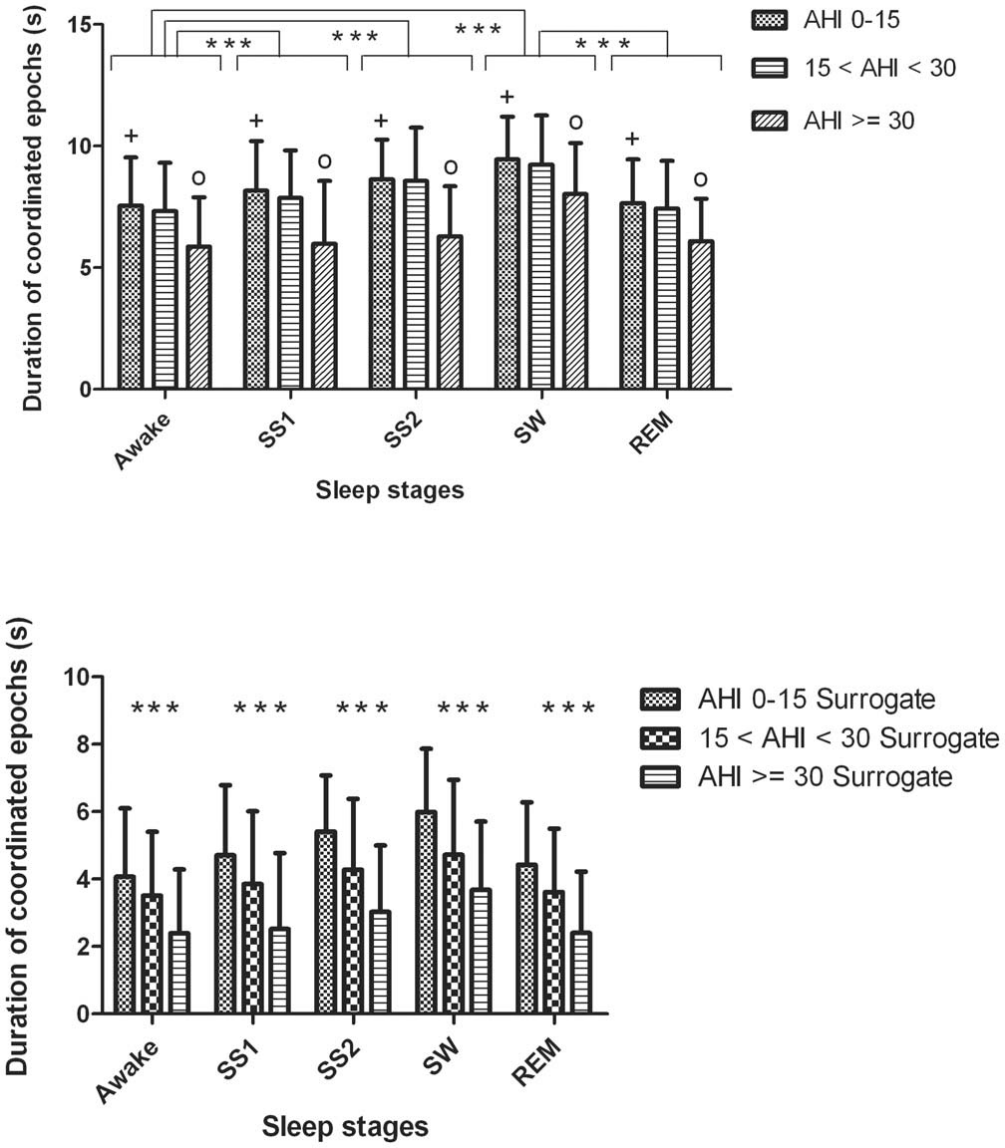
Group and sleep related comparison of average duration of coordinated epochs in seconds (AvDurCordn, mean ± SD) using original (upper panel) and surrogate data (lower panel) from abdominal signal. The circle indicates that the differences in AvDurCordn between the moderate and severe OSA groups are significant (*p*<0.01) for all the sleep stages. Similarly, the plus indicates the significant differences (*p*<0.001) in AvDurCordn between no/mild and severe OSA groups for all the sleep stages. *** (p<0.0001) indicates the significant differences in AvDurCordn between different sleep stages as well as the original and surrogate data.

Cardiorespiratory coordination was significantly associated with the degree of severity of OSA. Patients with severe OSA had a significantly lower percentage of heart rate - respiratory rate phase-locking states compared to patients with no or mild OSA (Figure 3, upper panel). The average duration of coordinated epochs was significantly decreased in patients with severe OSA compared to patients with moderate or no/mild OSA (Figure 4, upper panel).

### 2. Predominant phase locking ratios

4∶1 was the most frequent phase locking ratio observed during wake (in 50 subjects), during stage 2 sleep (55 subjects), during slow-wave sleep (56 subjects) and during REM sleep (47 subjects). The 5∶1 ratio was the second most frequently observed during wake, stage 2, slow-wave and REM sleep respectively in 26, 33, 47 and 35 subjects. The 3∶1 and 7∶2 ratios were further dominant phase locking ratios that were found in approximately 16% of the patients.

### 3. Apnea-hypopnea index effects on cardiorespiratory coordination

The AHI showed negative correlations with the mean RR interval (*r* = −0.244, *p*<0.001), percentage of coordination (%cordn) (*r* = −0.463, *p*<0.001) and average duration of coordinated epochs (AvDurCordn) (*r* = −0.437, *p*<0.001).

### 4. Sleep stage effects on cardiorespiratory coordination

**Figure 5:**
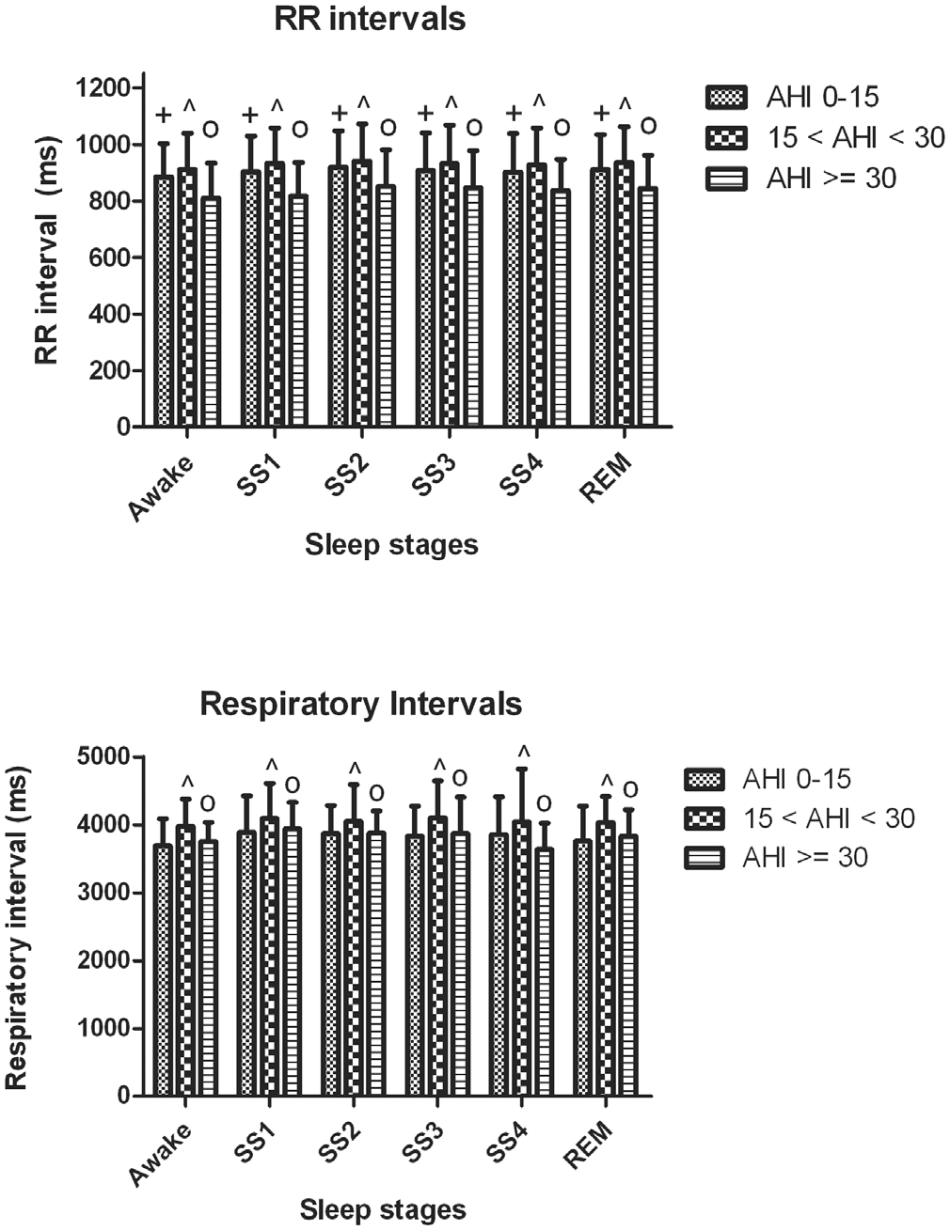
Group and sleep related comparison of RR interval and respiratory interval. The plus indicates that the differences in RR interval between the no/mild and severe OSA groups are significant (*p*<0.0001). Similarly, the circle and hat indicates the significance (*p*<0.0001) in RR interval or respiratory interval differences between moderate and severe or no/mild OSA groups respectively.

Mean heart rate and respiratory rate were not significantly different between sleep stages (see Figure 5). The percentage of coordination (%cordn) was nearly 50% higher during slow-wave sleep compared to REM sleep (18.7±7.7 vs. 11.2±4.3%, *p*<0.0001). The average duration of coordinated epochs (AvDurCordn) also depended on the sleep stage (Figure 4, upper panel). Post-hoc analysis revealed: i) prolongation of coordinated epochs in slow-wave sleep compared to REM sleep; and ii) prolongation of coordinated epochs in stage 1, stage 2 and slow-wave sleep compared to wakefulness. There was no significant difference in the dominance of phase locking ratios during different sleep stages (Tables 2 and 3).

### 5. Surrogate data

The percentage of coordination (%cordn) and average duration of coordinated epochs (AvDurCordn) was significantly decreased (*p*<0.001 and *p*<0.0001 respectively) after randomizing the order of the R wave occurrence (Figure 3 and 4, lower panels). Moreover, comparing the results obtained using surrogate data it was observed that they followed similar pattern and significance among the different AHI groups and sleep stages as that of the results obtained using original data. The %cordn and AvDurCordn, obtained using surrogate data, was significantly higher during slow-wave sleep compared to REM sleep (9.9±3.9 vs. 5.7±2.4%, *p*<0.0001 and 5.9±1.9 vs. 4.4±1.8 s, *p*<0.0001 respectively, in patients with no/mild OSA) (Figure 3 and 4, lower panels).

### 6. Age effects on cardiorespiratory coordination

Using the linear regression model for the 144 patients without heart disease, we found no significant association between age and percentage of coordination (%cordn). Likewise, age was not significantly associated with the average duration of coordinated epochs (AvDurCordn) and locking ratio. On the other hand, there was a significant positive correlation between the age and the mean RR interval (*r* = 0.188, *p*<0.01) and a significant positive correlation between age and mean respiratory time period (*r* = 0.242, *p*<0.001). To further investigate the age effect on heart rate and respiratory rate, the study group was divided into four arbitrary subgroups: <30 years, 30–45 years, 46–60 years and >60 years. The mean RR interval was significantly longer in patients above 60 years of age, compared to patients in the range of 30–45 years (967.92±88.55 vs. 877.01±114.32 ms). On the other hand, the mean respiratory time period was significantly higher in patients above 60 years of age, compared to patients less than 30 years of age (4051.32±173.67 vs. 3579.95±325.63 ms).

### 7. Gender effects on cardiorespiratory coordination

Analysing 144 patients without heart diseases, we observed that gender had no significant effect on RR interval (*r* = −0.072, *p*>0.1) or respiratory time period(*r* = 0.029, *p*>0.1). Males and females had similar patterns of coordination between heart rate and respiration. Considering ‘0’ as females and ‘1’ as males, a negative correlation was observed between gender and percentage of coordination (%cordn) (*r* = −0.180, *p*<0.01), between gender and duration of coordinated epochs (AvDurCordn) (*r* = −0.149, *p*<0.05) and between gender and locking ratio (*r* = −0.204, *p*<0.01).

### 8. BMI effects on cardiorespiratory coordination

Although BMI showed no correlation with percentage of coordination (%cordn) (*r* = −0.007, *p*>0.1) in the 144 patients without heart diseases, a significant correlation was observed between BMI and duration of coordinated epochs (*r* = −0.262, *p*<0.01). BMI showed significant negative correlations with both the mean RR interval (*r* = −0.343, *p*<0.001) the mean respiratory time period(*r* = −0.144, *p*<0.05). The association of heart rate and respiratory rate with BMI was further assessed by dividing the cohort into four groups based on the statistical categories of BMI values: underweight (<18.5 kg/m^2^), normal (18.5–25 kg/m^2^), overweight (25.1–30 kg/m^2^) and obese (>30 kg/m^2^). The RR interval and respiratory time period was significantly shorter in obese patients compared to patients with normal BMI (938.39±135.75 vs. 881.61±111.65 ms, *p*<0.001 and 3997.34±296.03 vs. 3888.53±355.231 ms, *p*<0.01). No significant difference in cardiorespiratory coordination was observed between the four BMI categories.

### 9. Effects of the respiratory signal source on the quantification of cardiorespiratory coordination

All results reported above were based on analysing the abdominal respiratory signal. To determine whether the origin of the respiratory signal (abdomen vs. thorax) affects our results, we compared the percentage of coordination (%cordn) and average duration of coordinated epochs (AvDurCordn) obtained from the abdominal trace with those obtained from the thorax (Tables 5 and 6). There was no significant difference in %cordn and AvDurCordn values obtained from thorax or abdomen (*p*>0.05).

**Table 5:** Percentage of coordination for different AHI groups and sleep stages using abdominal (ABDO) and thoracic (THOR) signals.

	*AHI≤15*		*15<AHI<30*		*AHI≥30*	
	ABDO	THOR	ABDO	THOR	ABDO	THOR
Awake	10.8±5.6%	8.3±4.5%	10.6±5.6%	7.9±3.8%	8.3±4.5%	6.3±3.6%
SS1	11.9±6.9%	10.7±5.5%	11.1±6.5%	9.4±4.4%	7.2±4.8%	7.8±4.5%
SS2	14.8±6.4%	13.7±6.1%	12.6±4.6%	11.9±4.7%	8.2±4.5%	8.3±4.2%
SW	18.7±7.8%	17.1±7.5%	14.4±4.8%	13.7±5.2%	13.3±5.4%	11.8±5.9%
REM	11.3±4.3%	10.1±4.7%	9.4±3.8%	8.7±3.8%	7.1±3.3%	6.9±4.6%

**Table 6:** Duration of coordinated epochs for different AHI groups and sleep stages using abdominal (ABDO) and thoracic (THOR) signals.

	*AHI≤15*		*15<AHI<30*		*AHI≥30*	
	ABDO	THOR	ABDO	THOR	ABDO	THOR
Awake	7.6±1.9 s	7.2±4.5 s	7.3±1.9 s	7.2±1.8 s	5.9±2.1 s	6.5±2.1 s
SS1	8.2±2.1 s	7.9±2.3 s	7.9±1.9 s	7.8±2.7 s	5.9±2.5 s	5.7±1.7 s
SS2	8.6±1.6 s	9.1±1.8 s	8.6±2.2 s	8.7±2.4 s	6.3±2.1 s	6.2±2.1 s
SW	9.5±1.8 s	9.7±1.9 s	9.2±2.1 s	9.4±2.4 s	8.1±2.1 s	7.9±2.6 s
REM	7.7±1.8 s	7.5±2.1 s	7.4±1.9 s	7.4±1.8 s	6.1±1.7 s	5.1±1.5 s

### 10. Correlation between percentage of coordination and heart rate, respiratory time period and high frequency power of heart rate

The mean RR-interval and respiratory time interval of the cohort was 906.1±126.5 ms and 3821.66±315.76 ms, respectively. The percentage of coordination (%cordn) showed no significant correlation with mean RR interval (*r* = 0.095, *p*>0. 1), respiratory rate (*r* = 0.015, *p*>0.1) or high frequency power of the HRV (logHF), computed as the logarithm of power in the frequency range 0.15–0.4 Hz, (*r* = 0.01, *p*>0.5). Average duration of coordinated epochs (AvDurCordn) was significantly correlated with mean RR interval (*r* = 0.383, *p*<0.001) and respiratory time interval (*r* = −0.492, *p*<0.001), but not with logHF (*r* = 0.11, *p*>0.1).

### 11. Effect of sleep stage and severity of OSA on HF power of heart rate

**Figure 6:**
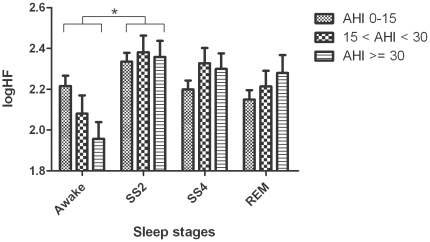
Group and sleep related comparisons of log transformed high frequency power (logHF) (mean ± SD). (* represents p<0.05).

The high frequency power component of HRV (logHF), i.e. the magnitude of RSA, was significantly different between awake and stage 2 sleep (Figure 6). The degree of OSA had no significant effect on the magnitude of high frequency power.

## Discussion

This study is the first to investigate the effect of OSA on cardiorespiratory coordination. Our major findings are: (1) the duration of phase-locking between cardiac and respiratory rhythms decreases with the severity of OSA; (2) the percentage of cardiorespiratory coordination and the duration of coordinated epochs is higher during slow-wave sleep compared with REM sleep; (3) 4∶1 is the most frequent phase locked ratio for cardiorespiratory coordination; (4) cardiorespiratory coordination is not affected by age or BMI.

The association between cardiac and respiratory rhythms has long been recognized. Respiratory sinus arrhythmia (RSA), i.e. subtle rhythmic heart rate accelerations/decelerations that oscillate around the respiratory frequency is a well-known phenomenon that has been observed in humans as well as in mammals. Although the physiological significance of RSA is yet to be established there is clinical evidence that reduced RSA is a prognostic indicator for cardiac mortality [32], [33]. Moderate exercise, on the other hand, has shown to increase the magnitude of RSA recorded at rest [34]. RSA is usually quantified by means of the high frequency power of HRV which is significantly influenced by breathing rate [35], [36]. It provides a basic surrogate, but completely lacks information on the respiratory system. We determined the effects of sleep stage and degree of OSA on high frequency power of HRV (figure 6). Although a significant difference in high frequency power was observed between awake and stage 2 sleep, the degree of OSA showed no effect on high frequency power. This suggests that cardiorespiratory coordination and RSA can be considered as two independent phenomena. However, according to Schafer et al, cardiorespiratory coordination can be reduced by an increase in RSA [17], [20].

Traditionally, the coupling between heart rate and respiration, termed as cardioventilatory coupling (CVC), has been studied based on the temporary alignment between the R waves of the ECG and inspiratory onsets; the plot used to study the temporary alignment being named as the RI plot. One of the recent studies by Tzeng *et al.* [37] suggested that, in addition to the RI plot, the respiratory period and the ratio of heart rate to respiratory rate should also be considered for accurate determination of CVC, since the presence of CVC on the RI plot was observed to be dependent on the ratio of heart rate to respiratory rate and hence would not accurately reflect the strength of coupling. The study also showed that arterial baroreceptors play a role in the process of CVC with very little contribution from vagal afferents and suggested CVC as a determinant of consecutive differences in respiratory period [37]. However, a recent study by Mangin et al showed that changes in breathing rate by mechanical ventilation can alter the chaotic properties of the coupling patterns and hence can alternatively be used in determining the CVC process [38]. In another study it was shown that CVC coupling is strongest when the low ventilatory frequency and short-term HRV is high [39].

In the present study we applied a different technique that assesses the interaction between heart and respiratory rates based on phase-looking and has some technical advantages over the traditional approach. Importantly, it does not require the detection of the inspiratory onset, which can be challenging, in particular when large amounts of data need to be processed, e.g. PSG recordings. Although the detection of inspiratory onset was not required for the study of cardiorespiratory coordination, it was used to determine the respiratory time period. The trade-off is that phase locking in general, independent of the phase between inspiratory onset and R wave, might not always in line with the physiological model. Despite of this shortcoming, there is evidence that the quantification of phase-looking might provide an useful tool to assess cardiorespiratory relationships [40]. Using a surrogate data approach, we found that the percentage of coordination and the average duration of coordinated epochs decrease significantly when the order of RR intervals is randomized. This suggests that cardiorespiratory coordination—as we have quantified and discussed below—indeed represent a deterministic feature of heart rate and respiration and is not a random observation. However, it also suggests that randomized RR intervals can show some coordination; up to 10% of the whole sleep duration, depending on the variability of the original RR interval series and probably also on the regularity of respiration. According to a recent study by Kenwright et al [41], the possibility of having an interaction between cardiac and respiratory oscillators is reduced by an increase in variation of respiratory frequency. It was shown that cardiorespiratory coordination was significantly decreased during exercise due to an increase in cardiac and respiratory frequencies as a result of greater demand for nutrients and oxygen from the blood by various body organs [41]. From our results it appears that heart rate and/or respiration are more regular during slow-wave sleep compared to REM sleep, since cardiorespiratory coordination is higher in slow-wave sleep compared to REM sleep, even when the order of RR intervals is random.

In our study we found that phase locking between heart and respiratory rhythms lasts for time intervals of on average 8.3 seconds in subjects with no/mild OSA. Although these phase-locked periods are rather short they account for up to 20% of the whole sleep duration. In accordance with a previous study [25], we found a profound sleep stage effect on cardiorespiratory coordination. Phase-locking occurs more often and for longer periods in slow-wave sleep compared to REM sleep. This might be the effect of less regular breathing patterns in REM sleep paralleled by sympathetic cardiac activation, making both rhythms more erratic and therefore phase-locking less likely. In line with that RSA has shown to increases during SW sleep [42]. REM sleep, characterized by irregular and high-frequency waves in the electroencephalogram is associated with a lack of synchrony between neuron firing rates, possibly due to desynchronized nervous activity [43], [44]. It has been suggested by Hamann et al [45] that cardiorespiratory coordination can be observed as long as the noise from higher brain regions affects the cardiac and respiratory oscillators is uncorrelated. However, during REM sleep, when the higher brain regions are more active, long-term correlated noise might be imposed on the two oscillators and thereby cause a reduction in the cardiorespiratory coordination [45]. It seems probable that the decrease in the amount of phase-locking between the cardiac and respiratory signals during REM sleep is caused by the desynchronized activity of the nervous system. Some of the earlier studies have also found changes in heart rate with the change in sleep stages [46], [47], [48]. However, in this study, the heart rate and respiratory rate showed no significant change with sleep stages. As almost one-third of our study group consisted of patients with heart disease their medication might partly explain the different heart rate behaviour.

In our study cardiorespiratory coordination was neither affected by age nor by BMI. These results are consistent with an earlier study by Bartsch et al [25]. However, in a recent study by Shiogai et al [49], it was reported that although cardiorespiratory coordination showed no correlation with age for males, it significantly increased with age for females. A different approach for detection of phases, Hilbert transform, used in this study, compared to marked events method used for cardiac phase detection by Shiogai et al [49], might be a possible cause of the variation in results. Similarly, the low sampling frequency and filtering approach to the respiratory signal used in this study could also be the contributing factors for the differences. According to another study, autonomic modulation of heart rate diminishes with age [50]. Interestingly, we did not observe a significant effect of age on cardiorespiratory coordination, suggesting that there is little association between HRV and the phase-locking between heart and respiratory rhythms. Also, in our study, a positive correlation between age and RR interval was unexpected as heart rate (reciprocal of RR interval) was reported to increase with age [47], [48].

The BMI has an effect on OSA and is also associated with an increased cardiac sympathetic tone [51], [52]. Patients with higher BMI are more likely to be affected by OSA. However, in our study, although OSA had an effect on the phase locking between heart rate and respiration, BMI showed no association with cardiorespiratory coordination. On the other hand, the respiratory time period had a negative correlation with BMI.

Autonomic modulation of heart rate is altered in OSA patients [10]. Our results indicate a significant increase in heart rate and respiratory rate in patients with moderate and severe OSA compared to patients with mild or no OSA. This increase in heart rate might be a result of a decrease in cardiac vagal outflow, of an increase in cardiac sympathetic outflow or both [53]. On the other hand cardiorespiratory coordination decreased significantly with the severity of OSA. This suggests that the amount of phase locking is influenced by autonomic control, which is affected by the repetitive obstructive episodes in OSA patients. Measures of cardiorespiratory coordination seem to be more sensitive to OSA than conventional HRV metrics alone, since they incorporate both, respiratory and cardiac domains.

The location of respiratory signal recording (abdomen vs. chest) might potentially influence cardiorespiratory coordination analysis. While the abdominal and thoracic excursions are approximately in phase during normal breathing, a higher difference in phase is typically observed during airway obstruction. It has been even suggested to use the phase difference between the two signals to detect sleep apnea [54]. For the purpose of this study we primarily used the respiratory signal obtained from the abdominal transducer. However, our analyses indicate that respiratory signals derived from the thorax can be equally used. There was no significant difference in the mean percentage of coordination for different AHI groups and sleep stages. Further, the sampling frequency has an influence on the accuracy of measurement of R-peaks, with a significant decrease in the amplitude of R-peak observed at a sampling rate of 125 Hz, compared to 500 Hz, but with no significant influence on the R-R interval [55]. The ECG sampling frequency for this study was relatively low (128 Hz). This, together with the technique used for quantifying synchronization and the short intervals of synchronized epochs considered as signature for cardiorespiratory coordination, could have influenced our results.

In conclusion, OSA perturbs the phase locking between cardiac and respiratory rhythms. The assessment of cardiorespiratory coordination may thus provide an ECG based screening tool for OSA.

## References

[1] Young T, Palta M, Dempsey J, Skatrud J, Weber S (1993). The occurrence of sleep-disordered breathing among middle-aged adults.. N Engl J Med.

[2] Roche F, Gaspoz JM, Court-Fortune I, Minini P, Pichot V (1999). Screening of obstructive sleep apnea syndrome by heart rate variability analysis.. Circulation.

[3] Tsuji H, Larson MG, Venditti FJ, Manders ES, Evans JC (1996). Impact of reduced heart rate variability on risk for cardiac events. The Framingham Heart Study.. Circulation.

[4] Hoffmann J, Grimm W, Menz V, Knop U, Maisch B (1996). Heart rate variability and major arrhythmic events in patients with idiopathic dilated cardiomyopathy.. Pacing Clin Electrophysiol.

[5] Sandercock GR, Brodie DA (2006). The role of heart rate variability in prognosis for different modes of death in chronic heart failure.. Pacing Clin Electrophysiol.

[6] Coruzzi P, Gualerzi M, Bernkopf E, Brambilla L, Brambilla V (2006). Autonomic Cardiac Modulation in Obstructive Sleep Apnea: Effect of an Oral Jaw-Positioning Appliance.. Chest.

[7] Wiklund U, Olofsson BO, Franklin K, Blom H, Bjerle P (2000). Autonomic cardiovascular regulation in patients with obstructive sleep apnoea: a study based on spectral analysis of heart rate variability.. Clin Physiol.

[8] Aydin M, Altin R, Ozeren A, Kart L, Bilge M (2004). Cardiac autonomic activity in obstructive sleep apnea: time-dependent and spectral analysis of heart rate variability using 24-hour Holter electrocardiograms.. Tex Heart Inst J.

[9] Roche F, Duverney D, Court-Fortune I, Pichot V, Costes F (2002). Cardiac interbeat interval increment for the identification of obstructive sleep apnea.. Pacing Clin Electrophysiol.

[10] Narkiewicz K, Montano N, Cogliati C, van de Borne PJH, Dyken ME (1998). Altered Cardiovascular Variability in Obstructive Sleep Apnea.. Circulation.

[11] Somers VK, Dyken ME, Clary MP, Abboud FM (1995). Sympathetic neural mechanisms in obstructive sleep apnea.. J Clin Invest.

[12] Baumert M, Smith J, Catcheside P, McEvoy RD, Abbott D (2008). Variability of QT interval duration in obstructive sleep apnea: an indicator of disease severity.. Sleep.

[13] Hayano J, Mukai S, Sakakibara M, Okada A, Takata K (1994). Effects of respiratory interval on vagal modulation of heart rate.. Am J Physiol Heart Circ Physiol.

[14] Song H-S, Lehrer PM (2003). The Effects of Specific Respiratory Rates on Heart Rate and Heart Rate Variability.. Appl Psychophysiol Biofeedback.

[15] Berntson GG, Bigger JT, Eckberg DL, Grossman P, Kaufmann PG (1997). Heart rate variability: origins, methods, and interpretive caveats.. Psychophysiology.

[16] Toledo E, Akselrod S, Pinhas I, Aravot D (2002). Does synchronization reflect a true interaction in the cardiorespiratory system?. Med Eng Phys.

[17] Schafer C, Rosenblum MG, Kurths J, Abel HH (1998). Heartbeat synchronized with ventilation.. Nature.

[18] Hoyer D, Hader O, Zwiener U (1997). Relative and intermittent cardiorespiratory coordination.. IEEE Eng Med Biol Mag.

[19] Bettermann H, Cysarz D, Van Leeuwen P (2002). Comparison of two different approaches in the detection of intermittent cardiorespiratory coordination during night sleep.. BMC Physiol.

[20] Schafer C, Rosenblum MG, Abel HH, Kurths J (1999). Synchronization in the human cardiorespiratory system.. Phys Rev E Stat Phys Plasmas Fluids Relat Interdiscip Topics.

[21] Censi F, Calcagnini G, Lino S, Seydnejad S, Kitney R (2000). Transient phase locking patterns among respiration, heart rate and blood pressure during cardiorespiratory synchronisation in humans.. Med Biol Eng Comput.

[22] Lotric MB, Stefanovska A (2000). Synchronization and modulation in the human cardiorespiratory system.. Physica A.

[23] Kotani K, Takamasu K, Ashkenazy Y, Stanley HE, Yamamoto Y (2002). Model for cardiorespiratory synchronization in humans.. Phys Rev E Stat Nonlin Soft Matter Phys.

[24] Cysarz D, Bettermann H, Lange S, Geue D, van Leeuwen P (2004). A quantitative comparison of different methods to detect cardiorespiratory coordination during night-time sleep.. Biomed Eng Online.

[25] Bartsch R, Kantelhardt JW, Penzel T, Havlin S (2007). Experimental Evidence for Phase Synchronization Transitions in the Human Cardiorespiratory System.. Phys Rev Lett.

[26] Mrowka R, Patzak A, Rosenblum M (2000). Quantitative analysis of cardiorespiratory synchronization in infants.. Int J Bifurc Chaos.

[27] Stefanovska A, Haken H, McClintock PV, Hozic M, Bajrovic F (2000). Reversible transitions between synchronization states of the cardiorespiratory system.. Phys Rev Lett.

[28] Hoyer D, Leder U, Hoyer H, Pompe B, Sommer M (2002). Mutual information and phase dependencies: measures of reduced nonlinear cardiorespiratory interactions after myocardial infarction.. Med Eng Phys.

[29] Leder U, Hoyer D, Sommer M, Baier V, Haueisen J (2000). Cardiorespiratory desynchronization after acute myocardial infarct.. Z Kardiol.

[30] Javorka M, Trunkvalterova Z, Tonhajzerova I, Javorkova J, Javorka K (2008). Short-term heart rate complexity is reduced in patients with type 1 diabetes mellitus.. Clin Neurophysiol.

[31] Rechtschaffen A, Kales A (1968). A manual of standarized terminology, techniques and scoring system for sleep stages in human subjects.

[32] Casolo GC, Stroder P, Signorini C, Calzolari F, Zucchini M (1992). Heart rate variability during the acute phase of myocardial infarction.. Circulation.

[33] Moser M, Lehofer M, Sedminek A, Lux M, Zapotoczky H (1994). Heart rate variability as a prognostic tool in cardiology. A contribution to the problem from a theoretical point of view.. Circulation.

[34] Meersman RE (1992). Respiratory sinus arrhythmia alteration following training in endurance athletes.. Eur J Appl Physiol.

[35] Hirsch JA, Bishop B (1981). Respiratory sinus arrhythmia in humans: how breathing pattern modulates heart rate.. Am J Physiol Heart Circ Physiol.

[36] Penttilä J, Helminen A, Jartti T, Kuusela T, Huikuri HV (2001). Time domain, geometrical and frequency domain analysis of cardiac vagal outflow: effects of various respiratory patterns.. Clin Physiol.

[37] Tzeng YC, Larsen PD, Galletly DC (2007). Mechanism of cardioventilatory coupling: insights from cardiac pacing, vagotomy, and sinoaortic denervation in the anesthetized rat.. Am J Physiol Heart Circ Physiol.

[38] Mangin L, Clerici C, Similowski T, Poon C-S (2009). Chaotic dynamics of cardioventilatory coupling in humans: effects of ventilatory modes.. Am J Physiol Regul Integr Comp Physiol.

[39] Tzeng Y, Larsen P, Galletly D (2003). Cardioventilatory coupling in resting human subjects.. Exp Physiol.

[40] Pereda E, Cruz DMDl, Vera LD, Gonzalez JJ (2005). Comparing generalized and phase synchronization in cardiovascular and cardiorespiratory signals.. IEEE Trans Biomed Eng.

[41] Kenwright D, Bahraminasab A, Stefanovska A, McClintock P (2008). The effect of low-frequency oscillations on cardio-respiratory synchronization.. Eur Phys J B Condensed Matter and Complex Systems.

[42] Brandenberger G, Buchheit M, Ehrhart J, Simon C, Piquard F (2005). Is slow wave sleep an appropriate recording condition for heart rate variability analysis?. Auton Neurosci.

[43] Guyton AC, Hall JE, Schmitt W, Gruliow R (2006). States of brain activity - sleep, brain waves, epilepsy, psychoses.. Textbook of medical physiology.

[44] Gottesmann C (1999). Neurophysiological support of consciousness during waking and sleep.. Prog Neurobiol.

[45] Hamann C, Bartsch RP, Schumann AY, Penzel T, Havlin S (2009). Automated synchrogram analysis applied to heartbeat and reconstructed respiration.. Chaos.

[46] Snyder F, Hobson JA, Morrison DF, Goldfrank F (1964). Changes in respiration, heart rate, and systolic blood pressure in human sleep.. J Appl Physiol.

[47] Penzel T, Kantelhardt JW, Lo CC, Voigt K, Vogelmeier C (2003). Dynamics of heart rate and sleep stages in normals and patients with sleep apnea.. Neuropsychopharmacology.

[48] Nalivaiko E, Catcheside PG, Adams A, Jordan AS, Eckert DJ (2007). Cardiac changes during arousals from non-REM sleep in healthy volunteers.. Am J Physiol Regul Integr Comp Physiol.

[49] Shiogai Y, Stefanovska A, McClintock PVE (2010). Nonlinear dynamics of cardiovascular ageing.. Physics Reports.

[50] Umetani K, Singer DH, McCraty R, Atkinson M (1998). Twenty-four hour time domain heart rate variability and heart rate: relations to age and gender over nine decades.. J Am Coll Cardiol.

[51] Ferguson KA, Ono T, Lowe AA, Ryan CF, Fleetham JA (1995). The Relationship Between Obesity and Craniofacial Structure in Obstructive Sleep Apnea.. Chest.

[52] Busetto L, Enzi G, Inelmen EM, Costa G, Negrin V (2005). Obstructive sleep apnea syndrome in morbid obesity: effects of intragastric balloon.. Chest.

[53] Narkiewicz K, Somers VK (2003). Sympathetic nerve activity in obstructive sleep apnoea.. Acta Physiol Scand.

[54] Varady P, Bongar S, Benyo Z (2003). Detection of airway obstructions and sleep apnea by analyzing the phase relation of respiration movement signals.. IEEE Trans Instrum Meas.

[55] Pizzuti GP, Cifaldi S, Nolfe G (1985). Digital sampling rate and ECG analysis.. J Biomed Eng.

